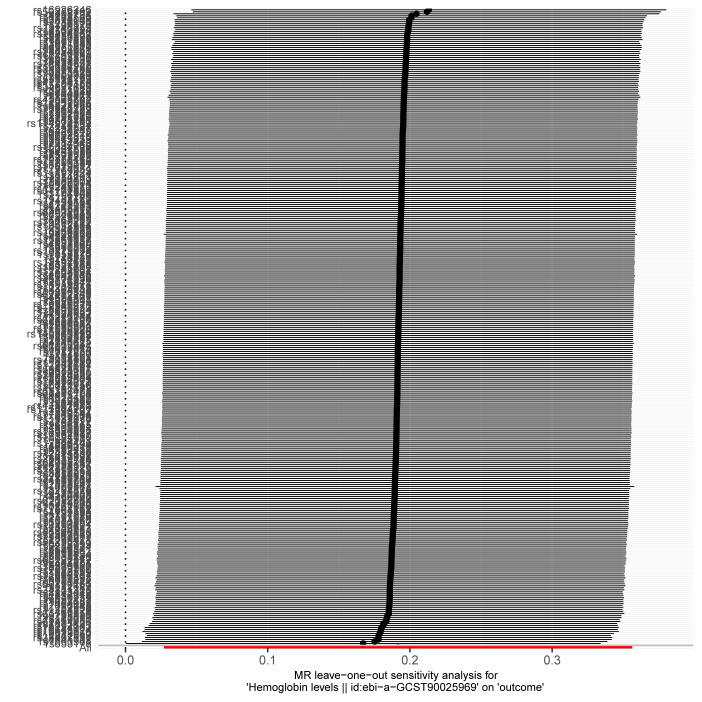# Identifying the association between hemoglobin levels and metabolic dysfunction-associated steatotic liver disease: An observational study and Mendelian randomization analysis

**DOI:** 10.1097/MD.0000000000048714

**Published:** 2026-05-08

**Authors:** Yang Qu, Guoxing Zhou, Feifei Yan

**Affiliations:** a Department of Hepatic Surgery, The First Affiliated Hospital of Harbin Medical University, Harbin, Heilongjiang, China; b Department of Radiology, First Affiliated Hospital, Heilongjiang University of Chinese Medicine, Harbin, Heilongjiang, China.

**Keywords:** causality, hemoglobin, Mendelian randomization analysis, metabolic dysfunction-associated steatohepatitis, National Health and Nutrition Examination Survey

## Abstract

Observational studies suggest a relationship between hemoglobin (Hb) levels and metabolic dysfunction-associated steatotic liver disease (MASLD), but its specific nature remains unclear. This study aimed to clarify the association through a National Health and Nutrition Examination Survey (NHANES) cross-sectional analysis and a 2-sample Mendelian randomization (MR) approach. We utilized NHANES data from 49,693 participants (2009–2018). Weighted multivariate logistic regression examined the Hb-MASLD association, with smoothed curve fitting and threshold effect analysis exploring nonlinear relationships. MR analyses assessed the observed causal relationship. Among 6165 NHANES participants, higher Hb levels were positively associated with MASLD risk (odds ratio = 1.15, 95% confidence interval = 1.08–1.23). Notably, when Hb concentrations exceeded 12.9 g/dL, MASLD risk significantly increased (odds ratio = 1.25, 95% confidence interval = 1.18–1.32). Two-sample MR analysis supported a causal relationship between elevated Hb levels and increased MASLD risk. Sensitivity analyses confirmed robustness (all *P* > .05). This study demonstrates that higher Hb levels are associated with an increased risk of MASLD, revealing potential nonlinear trends. MR analysis further confirmed this causal relationship, suggesting Hb levels could be a target for early intervention in preventing MASLD. These findings underscore the need for additional research to explore Hb’s role in MASLD development and to inform clinical guidelines.

## 1. Introduction

Metabolic dysfunction-associated steatotic liver disease (MASLD) is now the leading cause of chronic liver disease globally.^[1]^ It is characterized by a pathological state in which the accumulation of lipids in the liver cells exceeds 5% without the influence of secondary factors, leading to chronic liver damage. This condition ranges from uncomplicated hepatic steatosis to more advanced metabolic dysfunction-associated steatohepatitis. In a small number of patients, it can progress to cirrhosis and hepatocellular carcinoma.^[1,2]^ Under this new nomenclature, MASLD was referred to as metabolic dysfunction-associated fatty liver disease (MASLD).^[3]^ The impact of severe MASLD-related diseases is expected to more than double by 2030 compared with 2016, driven by increasing metabolic risk factors and an aging population.^[4,5]^ A recent large meta-analysis indicated that one-third of adults may have MASLD, with rates continuing to rise at an alarming pace.^[6]^ MASLD has become the primary cause of liver transplantation and hepatocellular carcinoma, imposing a significant public health burden annually.^[7,8]^

Previous studies have shown a relationship between hemoglobin (Hb) levels and the occurrence of MASLD.^[9,10]^ A phase II clinical trial also demonstrated that reducing Hb levels through phlebotomy improved the MASLD activity score,^[11]^ further confirming the close association between Hb and MASLD.

Despite these clinical observations, systematic evaluations of the relationship between Hb levels and the incidence of MASLD – the nomenclature recently updated from non-alcoholic fatty liver disease in accordance with the 2023 multi-society Delphi consensus – in large-population studies are still lacking. Specifically, 3 major literature gaps remain: first, a lack of large-scale, nationally representative cross-sectional studies to robustly validate this association; second, an absence of investigations into the potential nonlinear dose–response relationships and threshold effects between Hb levels and MASLD incidence; and third, a scarcity of genetic evidence to establish a definitive causal direction, which is necessary to overcome the residual confounding and reverse causality inherent in traditional epidemiological studies.

To address these gaps, we utilized the National Health and Nutrition Examination Survey (NHANES), which provides high-quality, large-scale, nationally representative data ideal for evaluating the complex, potentially nonlinear relationship between Hb levels and the risk of MASLD.^[12]^ Furthermore, to establish causality, we employed Mendelian randomization (MR) analysis. MR leverages the random distribution of alleles at conception to evaluate causal relationships,^[13]^ using genetic variants – typically single-nucleotide polymorphisms (SNPs) – as instrumental variables (IVs) for associated exposures,^[14,15]^ thereby mitigating the confounding factors often present in observational research.^[16,17]^

Therefore, this study aimed to bridge these specific gaps by clarifying both the observational association (including nonlinear trends) and the genetic causal relationship between Hb levels and MASLD. By integrating a large-scale NHANES cross-sectional analysis with a 2-sample MR approach, we sought to provide robust, multidimensional evidence to inform clinical guidelines for managing Hb levels in at-risk populations.

## 2. Methods

### 2.1. Overall study design

In this study, we conducted multivariable regression analysis using cross-sectional data from the NHANES to explore the relationship between Hb levels and MASLD. In addition, we used MR to investigate the causal relationship between Hb levels and MASLD. The findings from this study provide valuable insights into the causal relationship between Hb and MASLD, potential mechanisms, and preventive measures.

### 2.2. Data sources and study population

The NHANES is a comprehensive program conducted by the National Center for Health Statistics to collect health-related data from non-institutionalized US citizens. The NHANES comprises 5 main data collection components: demographics, diet, physical examination, laboratory results, and questionnaire data. The National Center for Health Statistics Research Ethics Review Board approved this survey, and all participants provided informed consent. The survey was conducted by a team of physicians, medical technicians, and health interviewers and included household interviews and health examinations. The NHANES data are publicly available on the Centers for Disease Control and Prevention website “www.cdc.gov/nchs/nhanes/,” providing valuable insights into the current health status of the US population. We used these survey data to investigate the potential association between Hb levels and the incidence of MASLD in adults in the United States.

We downloaded NHANES data from 5 cycles spanning 2009 to 2018, covering the years 2009–2010, 2011–2012, 2013–2014, 2015–2016, and 2017–2018, involving 49,693 individuals. Among these, 28,835 were aged 20 and above. After excluding individuals with missing Hb data and the variables required to calculate the US Fatty Liver Index (US-FLI), including waist circumference, fasting glucose, fasting insulin, and gamma-glutamyl transferase, 11,812 participants were included.

**Figure 1.**
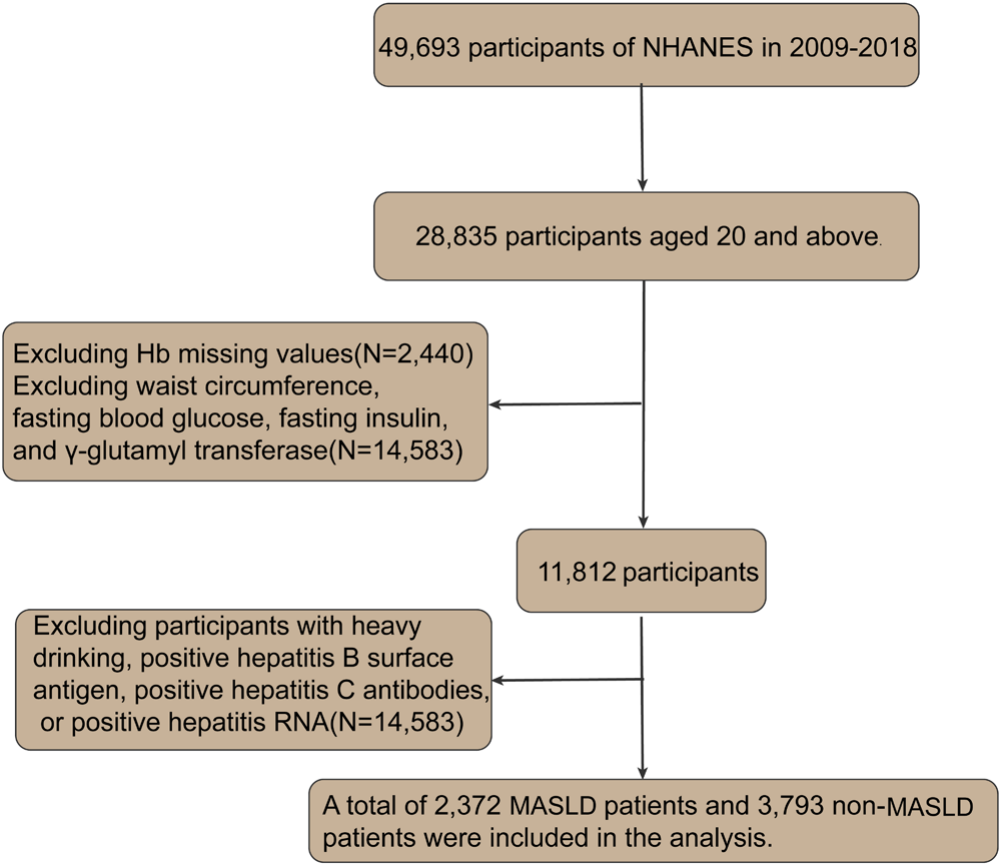
Flowchart of the study. Hb = hemoglobin, MASLD = metabolic dysfunction-associated steatotic liver disease, NHANES = National Health and Nutrition Examination Survey.

We further excluded individuals who were heavy drinkers (defined as men consuming ≥2 drinks per day or women consuming ≥1 drink per day) and those who tested positive for hepatitis B surface antigen, hepatitis C antibodies, or hepatitis RNA. The final analysis included 2372 patients with MASLD and 3793 without MASLD. The procedure for selecting study participants is depicted in detail in Figure 1.

### 2.3. Definition of MASLD

Fatty liver was defined using the US-FLI, which is calculated as follows: US-FLI = e^y^/(1 + e^y^) × 100, where y = −0.8073 × non-Hispanic Black + 0.3458 × Mexican American + 0.0093 × age + 0.6151 × log_e_ (gamma-glutamyl transferase) + 0.0249 × waist circumference + 1.1792 × log_e_ (insulin) + 0.8242 × log_e_ (glucose) − 14.7812. The variables for “non-Hispanic black” and “Mexican American” were coded as 1 if the participant identified with that ethnicity and 0 otherwise.^[18]^ Fatty liver is defined by a US-FLI score of ≥30, as recommended.^[19–21]^ MASLD is identified by a US-FLI score of ≥30, discounting other established reasons for chronic liver disease. These include viral hepatitis, indicated by positive markers such as hepatitis B surface antigen, hepatitis C antibody, or hepatitis C RNA, and significant alcohol consumption (≥2 drinks per day for men or ≥1 drink per day for women).

In alignment with the 2023 multi-society Delphi consensus, the nomenclature has been updated from non-alcoholic fatty liver disease to MASLD. The updated diagnostic criteria for MASLD require the presence of hepatic steatosis in conjunction with at least one cardiometabolic risk factor. In this study, hepatic steatosis was identified using a US-FLI score of ≥30. Because the calculation of the US-FLI inherently incorporates crucial metabolic parameters – specifically waist circumference, fasting glucose, and fasting insulin – all participants identified as having steatosis in our cohort concurrently possessed at least 1 cardiometabolic risk factor. Therefore, our selected study population strictly fulfills the contemporary diagnostic criteria for MASLD, ensuring both methodological robustness and clinical relevance.

These exclusions eliminate secondary causes of hepatic steatosis and prevent viral confounding of systemic iron metabolism and Hb levels, thereby ensuring our cohort strictly represents primary MASLD.

### 2.4. Assessment of covariates

We examined a range of covariates as potential confounders across various domains. Sociodemographic factors included age groups (20–39, 40–59, and >59 years), sex (male or female), race/ethnicity (non-Hispanic White, non-Hispanic Black, Hispanic, and others), educational level (below high school, high school, college or Associate of Arts degree, college graduate or above), and poverty-income ratio (<1.0, 1.0–3.0, >3.0).

Lifestyle factors included total caloric intake (calculated as the average caloric intake from dietary data on the first and second days), moderate work activity, and smoking status (yes or no).

Health status indicators included body mass index (BMI) (<30, ≥30), hypertension (yes or no), diabetes (yes or no), and high cholesterol (yes or no).

Laboratory indicators included Hb, alanine aminotransferase, aspartate aminotransferase, total cholesterol, triglycerides, low-density lipoprotein cholesterol, and high-density lipoprotein cholesterol.

### 2.5. MR analysis

#### 2.5.1. Study design

We employed a 2-sample MR analysis to test the causal relationship between genetically predicted Hb levels and MASLD. All studies included in the analysis received ethical approval from the relevant academic ethics committees, and participants provided informed consent. As this study did not utilize original data, specific ethical approval was not required.

The genetic variants employed for MR analysis had to meet 3 essential assumptions: relevance: the genetic variants are significantly associated with Hb levels; independence: the genetic variants are not linked to confounding factors; and exclusion restriction: the genetic variants influence the outcome solely through their effect on Hb concentration.^[22]^

#### 2.5.2. GWAS data source

The Genome-Wide Association Studies (GWAS) data for Hb levels were acquired from the IEU OPEN GWAS project, based on the study by Barton et al, which included a sample size of 445,373 Europeans and 4,234,826 available SNPs. The GWAS data for MASLD were sourced from the FinnGen R10 association database, which comprises 450,727 Europeans (2568 cases and 448,159 controls). Detailed information about the data is provided in Table S1, Supplemental Digital Content.

#### 2.5.3. Selection of IVs

Initially, we adopted a genome-wide significance threshold of 5E-8, excluding SNPs with linkage disequilibrium (*r*^2^ > 0.001 or a clustering distance within 10,000 kb) to ensure independence. Palindromic and synonymous SNPs were systematically excluded to minimize the bias caused by linkage or allele coding. To identify potentially weak IVs, we calculated the *F*-statistic (*F* = *R*^2^ [N − 2]/[1 − *R*^2^]) for each SNP and set the threshold at *F* > 10 to exclude weak IVs.^[23]^ MR-Steiger tests were conducted to assess the role of IVs in explaining exposure-outcome changes and to verify the directionality of causal relationships.

#### 2.5.4. MR analysis

To evaluate the link between Hb levels and MASLD, we used a random-effects inverse-variance weighted (IVW) model as the main MR analysis method.^[24]^ IVW is a widely used method that combines the Wald ratios for each SNP to generate overall estimates. In addition, we performed 4 complementary analyses: weighted median, Mendelian randomization Egger (MR-Egger), weighted mode, and simple mode. MR-Egger intercept analyses were conducted to assess horizontal pleiotropic effects.^[25]^ The Cochran *Q* test, based on the IVW and MR-Egger methods, was used to assess heterogeneity; a *P* value >.05 indicates no significant heterogeneity. We also used the MR-Egger intercept to estimate the level of genetic variation associated with pleiotropy (*P* < .05), suggesting potential pleiotropy.^[26]^ To ensure the robustness of the study, leave-one-out analysis was conducted to systematically evaluate the impact of each excluded SNP on the overall causal estimates. Analyses were performed using the TwoSample MR (0.6.6) software package.

### 2.6. Statistical analysis

To ensure an accurate estimation of the sampling error and representativeness of the entire US population, we applied sample weights. Hb concentrations were categorized into 4 quartiles: Q1 (<13.2 g/dL), Q2 (13.2–14.1 g/dL), Q3 (14.2–15.1 g/dL), and Q4 (≥15.2 g/dL). Statistical analyses were performed using R version 4.2.3 and EmpowerStats. Continuous variables were presented as weighted averages along with 95% confidence intervals (CIs), while categorical variables were represented as weighted percentages with corresponding 95% CIs. Weighted linear regression was employed to evaluate differences in continuous variables among groups, while weighted chi-square tests were utilized for categorical variables.

Weighted multivariate logistic regression was used to analyze the relationship between Hb levels and MASLD, with results reported as odds ratios (ORs) and 95% CIs.

To control for confounding factors, 3 regression models were used: an unadjusted model; a model adjusted for sex, age, and race; and a model adjusted for education level, poverty-income ratio, physical activity, total caloric intake, smoking, BMI, hypertension, diabetes, high cholesterol, alanine aminotransferase, aspartate aminotransferase, total cholesterol, triglycerides, low-density lipoprotein cholesterol, and high-density lipoprotein cholesterol levels.

To evaluate the potential risk of multicollinearity among the extensive metabolic variables included in the fully adjusted model (model 3), the variance inflation factor was calculated. All covariates demonstrated a variance inflation factor of <5, indicating no severe collinearity that would distort the model estimates or mask the independent effect of Hb.

## 3. Results

### 3.1. Baseline characteristics

This analysis included 6165 individuals, with 48.64% being male and 77.79% identifying as non-Hispanic White. Among the participants, 2372 individuals (36.47%) were diagnosed with MASLD. After further weighting, the average age of the cohort was 45.73 years, and the mean Hb concentration was 14.16 g/dL. Table 1 presents the weighted baseline characteristics of the participants. We found that patients with MASLD were typically older, had a higher proportion of males, and were predominantly non-Hispanic whites.

**Table 1:** Characteristics of the study population (n = 6156).

Characteristic	Overall	MASLD, N = 2372	Non-MASLD, N = 3793	*P* value
Age (yr)	45.73 (46.83, 48.07)	50.47 (49.59, 51.36)	45.73 (44.96, 46.49)	<.001
Age group (%)				
20–39	36.55 (35.19, 37.93)	36.64 (34.03, 39.33)	36.50 (34.75, 38.29)	.3519
40–59	35.58 (34.17, 37.02)	36.85 (34.31, 39.45)	34.86 (32.95, 36.82)	
>59	27.87 (26.46, 29.33)	26.52 (24.08, 29.11)	28.64 (26.82, 30.53)	
Gender, n (%)				
Male	48.64 (47.37, 49.92)	55.49 (52.45, 58.48)	44.75 (43.10, 46.40)	<.001
Female	51.36 (50.09, 52.63)	44.51 (41.52, 47.55)	55.25 (53.60, 56.90)	
Race/ethnicity, n (%)				
Non-Hispanic White	77.79 (75.30, 81.09)	76.24 (73.17, 79.05)	78.67 (76.12, 81.01)	<.001
Non-Hispanic Black	10.22 (8.58, 12.13)	11.33 (9.32, 13.70)	9.58 (8.03, 11.41)	
Hispanic	6.79 (5.65, 8.14)	8.18 (6.54, 10.17)	6.00 (4.91, 7.32)	
Other	5.21 (4.44,6.09)	4.26 (3.31, 5.46)	5.75 (4.78, 6.89)	
Education levels, n (%)				
Below high school or high school	58.41 (56.90, 59.92)	60.07 (57.26, 62.82)	57.47 (55.46, 59.46)	.053
Some college or AA degree	35.56 (34.11, 37.04)	35.10 (32.58, 37.71)	35.82 (33.95, 37.73)	
College graduate or above	6.02 (5.36, 6.76)	4.83 (3.85, 6.04)	6.70 (5.79, 7.75)	
Family income-poverty ratio, n (%)				
<1.0	18.75 (17.41, 20.18)	18.90 (16.97, 21.00)	18.67 (17.30, 20.42)	.933
1.0–3.0	40.32 (38.67, 41.99)	40.57 (37.68, 43.52)	40.18 (38.13, 42.26)	
>3.0	40.93 (39.17, 42.71)	40.53 (37.96, 43.16)	41.16 (38.84, 43.51)	
Smoking status, n (%)				
Yes	47.41 (45.66, 49.16)	47.96 (45.33, 50.60)	47.10 (44.83, 49.37)	.619
No	52.59 (50.84, 54.34)	52.04 (49.40, 54.67)	52.90 (50.63, 55.17)	
Moderate work activity				
Yes	43.04 (41.35, 44.75)	44.58 (42.19, 47.00)	42.16 (40.04, 44.31)	.110
No	56.97 (55.25, 58.65)	55.42 (53.00, 57.81)	57.84 (55.69, 59.96)	
BMI, kg/m^2^	29.70 (29.44, 29.95)	29.79 (29.37, 30.22)	29.64 (29.33, 29.95)	.558
BMI (%)				
<30	59.25 (57.59, 60.89)	60.29 (57.44, 63.06)	58.66 (56.55, 60.75)	.367
≥30	40.75 (39.11, 42.41)	39.71 (36.94, 42.56)	41.34 (39.25, 43.45)	
Total energy, kcal	2108.18 (2079.50, 2136.86)	2084.45 (2046.59, 2122.32)	2121.67 (2081.90, 2161.44)	.191
Hypertension, n (%)				
Yes	35.17 (33.54, 36.84)	36.44 (33.44, 39.54)	34.45 (32.47, 36.48)	.286
No	65.56 (65.13, 67.96)	63.56 (60.46, 66.56)	65.55 (63.52, 67.53)	
High cholesterol, n (%)				
Yes	33.44 (32.04, 34.87)	34.47 (32.14, 36.88)	32.85 (31.05, 34.70)	.290
No	66.56 (65.13, 67.96)	65.53 (63.12, 67.86)	67.15 (65.30, 68.95)	
Diabetes, n (%)				
Yes	10.93 (9.95, 11.99)	10.50 (8.99, 12.23)	11.17 (9.86, 12.64)	.548
No	89.07 (88.016, 90.05)	89.50 (87.77, 91.01)	88.83 (87.36, 90.14)	
Hb	14.35 (14.29, 14.41)	14.55 (14.46, 14.64)	14.24 (14.17, 14.30)	<.001
ALT, U/L	24.81 (24.25, 25.37)	24.67 (23.84, 25.49)	24.89 (24.14, 25.64)	.696
AST, U/L	25.43 (24.66, 26.20)	25.89 (24.34, 27.44)	25.17 (24.31, 26.03)	.434
TC, mg/dL	190.57 (189.29, 191.85)	191.75 (189.45, 194.05)	189.90 (188.21, 191.59)	.232
TG, mg/dL	126.39 (123.07, 129.71)	124.88 (120.86, 128.90)	127.25 (122.62, 191.87)	.447
LDL, mg/dL	112.52 (111.38, 113.66)	113.51 (111.49, 1151.53)	111.95 (110.53, 113.38)	.231
HDL, mg/dL	54.23 (53.77, 54.70)	54.73 (54.04, 55.41)	53.95 (53.27, 54.62)	.143

AA = Associate of Arts, ALT = alanine aminotransferase, AST = aspartate aminotransferase, BMI = body mass index, Hb = hemoglobin, HDL = high-density lipoprotein cholesterol, LDL = low-density lipoprotein cholesterol, MASLD = metabolic dysfunction-associated steatotic liver disease, NHANES = National Health and Nutrition Examination Survey, TC = total cholesterol, TG = triglyceride.

### 3.2. Association between Hb concentration and MASLD

**Figure 2.**
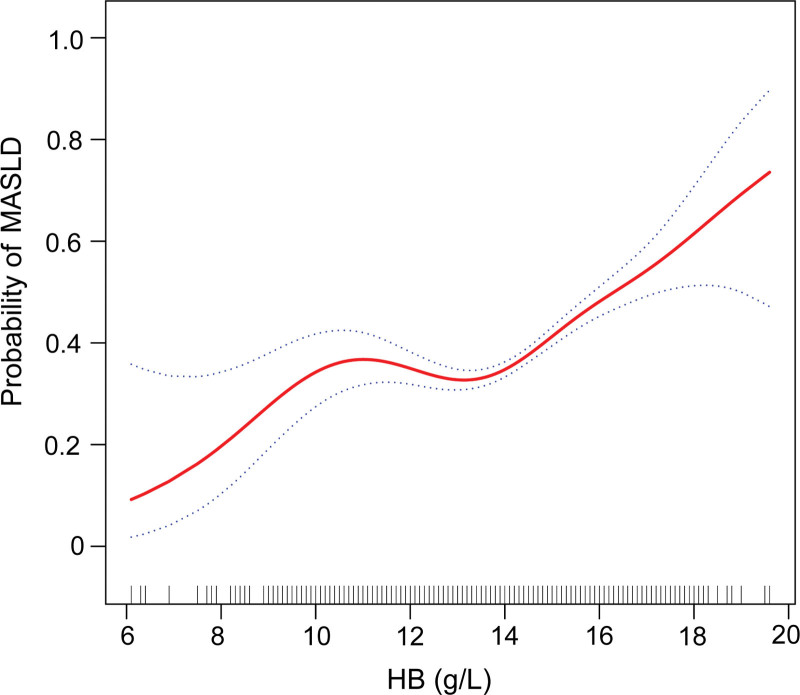
Smooth curve fitting of the relationship between Hb concentrations and MASLD probability. Hb = hemoglobin, MASLD = metabolic dysfunction-associated steatotic liver disease.

The results showed a significant positive correlation between Hb concentration and MASLD. After adjusting for all covariates (model 3), the weighted OR and their 95% CI for MASLD across different quartiles of Hb concentration (Q1–Q4) were as follows: 1.00 (reference group), 1.01 (0.83, 1.23), 1.09 (0.87, 1.37), and 1.64 (1.28, 2.11), with a trend test *P* value of <.001 (Table 2). When Hb was analyzed as a continuous variable, the association remained significant, with each unit increase in Hb corresponding to a 15% relative increase in the prevalence of MASLD (OR = 1.15, 95% CI = 1.08–1.23; Table 2). Smoothed curve fitting revealed a nonlinear relationship between Hb levels and MASLD (Fig. 2). Further threshold effect analysis identified a turning point at a Hb concentration of 12.9 g/dL (likelihood ratio, *P* < .05). Below this threshold, there was no significant association between Hb concentration and MASLD (OR = 0.96; 95% CI = 0.87–1.06). However, when Hb concentration exceeded 12.9 g/dL, each additional unit was associated with a 25% increase in MASLD incidence (OR = 1.25, 95% CI = 1.18–1.32; Table 3).

**Table 2:** ORs (95% CIs) for MASLD based on Hb concentrations, weighted.

Serum albumin	Model 1			Model 2			Model 3		
	OR	95% CI	*P* value	OR	95% CI	*P* value	OR	95% CI	*P* value
Hb*	1.17	1.12–1.23	<.001	1.16	1.09–1.23	<.001	1.15	1.08–1.23	<.001
Hb†									
Q1	1.00	–	–	1.00	–	–	1.00	–	–
Q2	1.01	0.84–1.23	.846	1.02	0.83–1.24	.870	1.01	0.83–1.23	.922
Q3	1.14	0.93–1.40	.214	1.09	0.87–1.37	.445	1.09	0.87–1.37	.459
Q4	1.75	1.47–2.08	<.001	1.65	1.29–2.12	<.001	1.64	1.28–2.11	<.001
*P* for trend			<.001			<.001			<.001

Model 1: non-adjusted.

Model 2: adjusted for sex, age, and race.

Model 3: adjusted for sex, age, race, education, PIR, BMI, physical activity, smoking, total caloric intake, hypertension, high cholesterol, diabetes, ALT, AST, TC, TG, LDL, and HDL.

CI = confidence interval, ALT = alanine aminotransferase, AST = aspartate aminotransferase, BMI = body mass index, Hb = hemoglobin, HDL = high-density lipoprotein cholesterol, LDL = low-density lipoprotein cholesterol, MASLD = metabolic dysfunction-associated steatotic liver disease, NHANES = National Health and Nutrition Examination Survey, OR = odds ratio, PIR = poverty-income ratio, TC = total cholesterol, TG = triglyceride.

* Each 1-unit increase in serum Hb concentrations.

† Q1: <13.2 g/dL; Q2: 13.2–14.1 g/dL; Q3: 14.2–15.1 g/dL; Q4: ≥15.2 g/dL.

**Table 3:** Threshold effect analysis of Hb concentrations on MASLD incidence.

	Adjusted OR (95% CI), *P* value*
Fitting by the standard linear model	1.16 (1.11–1.21), <.001
Fitting by the 2-piecewise linear model	
Inflection point	12.9 g/L
Serum albumin concentrations < 12.9 g/dL	0.96 (0.87–1.06), .428
Serum albumin concentrations ≥ 12.9 g/dL	1.25 (1.18–1.32), <.001
*P* for log-likelihood ratio	<.001

ALT = alanine aminotransferase, AST = aspartate aminotransferase, BMI = body mass index, CI = confidence interval, Hb = hemoglobin, HDL = high-density lipoprotein cholesterol, LDL = low-density lipoprotein cholesterol, MASLD = metabolic dysfunction-associated steatotic liver disease, OR = odds ratio, PIR = poverty-income ratio, TC = total cholesterol, TG = triglyceride.

* Adjusted for sex, age, race, education, PIR, BMI, physical activity, total energy intake, smoking, hypertension, high cholesterol, diabetes, ALT, AST, TC, TG, LDL, and HDL.

**Figure 3.**
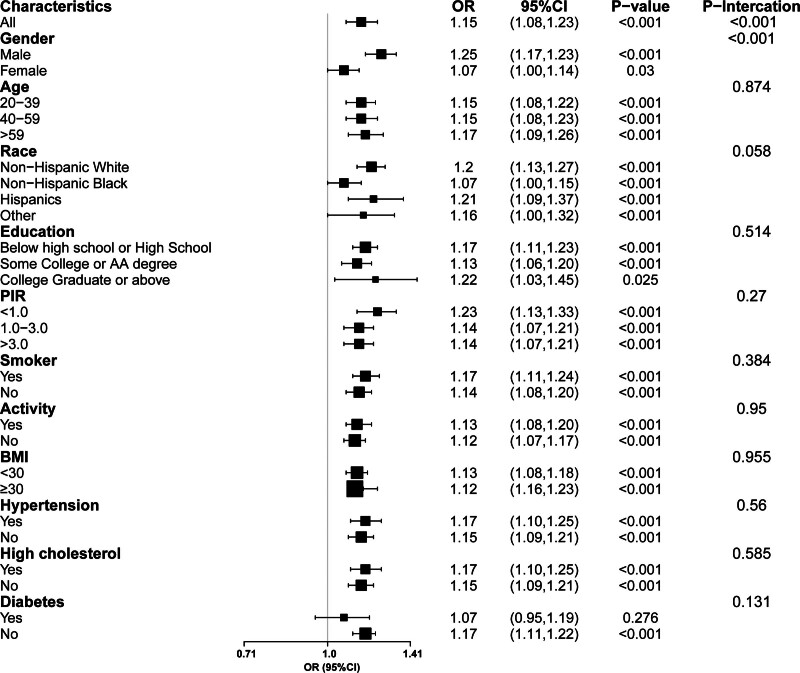
The subgroup analysis of the association between hemoglobin concentrations and MASLD incidence. The analysis is adjusted for sex, age, race, education, PIR, BMI, physical activity, smoking, total energy intake, hypertension, diabetes, hyperlipidemia, ALT, AST, TC, TG, LDL, and HDL. OR and 95% CI represent the odds ratio and confidence interval for each 1-unit increase in hemoglobin concentrations. ALT = alanine aminotransferase, AST = aspartate aminotransferase, BMI = body mass index, HDL = high-density lipoprotein cholesterol, LDL = low-density lipoprotein cholesterol, MASLD = Metabolic dysfunction-associated steatotic liver disease, PIR = poverty-income ratio, TC = total cholesterol, TG = triglyceride.

In addition, we conducted subgroup analyses after adjusting for factors such as sex, age, BMI, smoking, alcohol consumption, hypertension, and diabetes (Fig. 3). The results showed that the negative correlation between Hb concentration and MASLD prevalence was significantly stronger in men than in women (men: OR = 1.25, 95% CI = 1.17–1.23; women: OR = 1.07, 95% CI = 1.00–1.14; *P* interaction < .001).

### 3.3. MR analysis: causal relationships between Hb and MASLD risk

Given the significant positive correlation between Hb levels and MASLD risk identified in our multivariate regression analysis, we proceeded with MR analysis to explore the causal link between Hb and MASLD. After thorough selection, 326 SNPs were identified (Table S2, Supplemental Digital Content). Using the IVW method, we observed that genetically predicted Hb levels are associated with an increased risk of MASLD (OR = 1.21 [95% CI = 1.03–1.43], *P* = .022). The MR-Egger method produced similar findings (OR = 1.47 [1.07–2.03], *P* = .018). However, the weighted median, simple mode, and weighted mode methods did not indicate a causal relationship.

**Figure 4.**
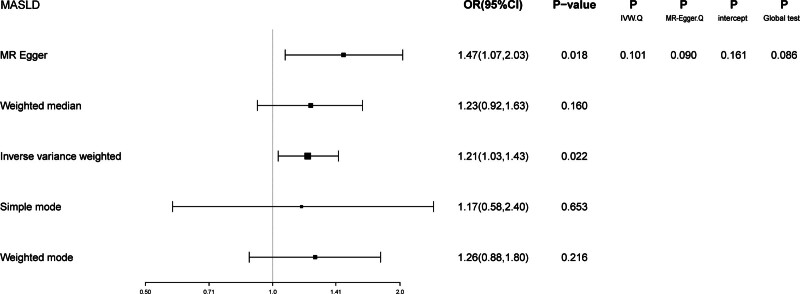
Forest plot and sensitivity analysis results from the MR study on the effects of Hb on MASLD. CI = confidence interval, Hb = hemoglobin, IVW = inverse-variance weighted, MASLD = metabolic dysfunction-associated steatotic liver disease, MR = Mendelian randomization, OR = odds ratio.

Moreover, the consistency of the results across various supplementary MR methods, both in terms of direction and magnitude of the causal effect, reinforces the reliability of our findings. Figure 4 illustrates the effects of MASLD exposure assessed through different MR methods. The scatter plot shows the relationship between Hb levels and MASLD risk (Fig. S1, Supplemental Digital Content), while the forest plot displays the estimated impact of each SNP on MASLD (Fig. S2, Supplemental Digital Content). We also conducted heterogeneity and horizontal pleiotropy analyses, which showed no signs of heterogeneity or horizontal pleiotropy (all *P* > .05; Fig. 4). The symmetry of the funnel plot further supports these conclusions (Fig. S3, Supplemental Digital Content). In addition, leave-one-SNP-out analysis confirmed that the causal relationship between Hb levels and MASLD was not influenced by any single SNP (Fig. S4, Supplemental Digital Content). Collectively, these results indicate that our findings are robust and reliable.

## 4. Discussion

As far as we know, this is the first comprehensive analysis that integrates genetic and large-scale observational data to investigate the association between Hb levels and the prevalence of MASLD. Our study demonstrates a significant positive correlation between Hb concentration and the prevalence of MASLD. Furthermore, using the 2-sample MR method, we found evidence suggesting a causal relationship between Hb levels and MASLD, indicating that Hb may be a modifiable risk factor for MASLD.

MASLD is defined as the excessive buildup of lipids in liver cells, often linked to insulin resistance, and occurs without significant alcohol intake. Prior research has underscored the significant association between Hb levels and the onset of MASLD. Notably, a phase II clinical trial showed that reducing Hb levels through phlebotomy improved MASLD activity scores. These findings indicate that Hb may contribute to the onset of MASLD. Our study further revealed a significant correlation between higher Hb levels and increased MASLD prevalence based on a comprehensively adjusted model from a large, population-based cross-sectional study. This discovery not only supports prior clinical findings but also reinforces the broad applicability of Hb in the occurrence and diagnosis of MASLD.

Surprisingly, our research also uncovered a nonlinear relationship between Hb and MASLD. Specifically, we observed a significant increase in MASLD incidence when Hb concentrations were 12.9 g/dL or higher, while no clear association was found below this threshold. Identifying a 12.9 g/dL threshold offers epidemiological value but requires cautious clinical interpretation as it falls within normal physiological ranges (particularly for women). Rather than a rigid diagnostic boundary, it likely represents a pathophysiological inflection point. High-normal Hb levels may reflect underlying insulin resistance, subclinical systemic inflammation, and mild hemoconcentration. These shifts increase blood viscosity, exacerbate hepatic microcirculatory resistance, and trigger oxidative stress, directly promoting hepatocyte injury. Consequently, Hb approaching 12.9 g/dL may serve as a composite clinical indicator of these converging inflammatory and lipotoxic mechanisms in MASLD pathogenesis.

In the subgroup analysis, we noted a notable interaction between Hb levels and sex, indicating that the effect of Hb on MASLD risk may vary depending on sex. In males, elevated Hb levels were more strongly linked to a higher risk of MASLD. We speculate that this gender discrepancy might be related to the protective effects of estrogen in premenopausal women, which can improve lipid metabolism and reduce hepatic fat accumulation.^[27,28]^ However, because our extracted NHANES dataset lacks specific hormonal data, this estrogen-driven mechanism remains a speculation. Future longitudinal studies incorporating comprehensive endocrine profiling are required to definitively validate this hypothesis. In the subgroup analysis of diabetic patients, the association between Hb and MASLD was found to be nonsignificant. Possible reasons for this include insufficient sample size: the sample size of diabetic patients may be too small to detect a significant association, especially in subgroup analyses; confounding factors: diabetic patients may have other metabolic abnormalities (such as insulin resistance and hyperglycemia) that could obscure the relationship between Hb and MASLD; and physiological mechanisms: diabetes may influence the metabolism or function of Hb, altering its relationship with hepatic fat metabolism. However, the association between Hb and MASLD is complex, making causal inferences challenging. Therefore, in addition to conducting a nationally representative observational study, we employed MR methods to elucidate the causal link between Hb levels and MASLD. Drawing from MR findings in European populations, our understanding of the positive causal relationship between Hb levels and MASLD has deepened. In addition to the primary IVW method, various supplementary MR approaches yielded consistent results. Sensitivity analyses further validated the strength and dependability of this positive association. This study is the first to reveal a link between Hb concentration and MASLD in patients, addressing a significant gap in the literature on MASLD. While the specific mechanisms remain unclear, potential explanations may include several factors.

First, higher Hb levels are often associated with higher iron levels.^[29,30]^

Approximately one-third of patients with MASLD show signs of iron overload, which is considered a key factor in triggering MASLD.^[31–33]^ Iron levels in the body are regulated by hepcidin, a peptide consisting of 25 amino acids. Hepcidin decreases iron absorption in the intestine and inhibits iron recycling from macrophages, leading to lower levels of iron in the bloodstream. Patients with MASLD/metabolic dysfunction-associated steatohepatitis often have abnormally low levels of hepcidin, which may promote iron uptake and increase the risk of iron overload.^[34]^ Although our extracted NHANES dataset lacked direct markers of iron metabolism such as ferritin, transferrin saturation, and hepcidin to empirically confirm this in our cohort, previous literature strongly supports this mechanistic link. Chronic low-grade inflammation inherent to MASLD can further impair hepcidin production, leading to a bidirectional cycle of iron accumulation, exacerbated insulin resistance, and compensatory erythropoiesis, which may clinically manifest as high-normal Hb levels. Simultaneously, iron overload can increase ferritin levels by enhancing the phagocytosis of ferritin and lipids via autophagy mechanisms.^[35]^ Once iron accumulates in the liver, it generates reactive oxygen species through the Haber–Weiss and Fenton reactions, leading to oxidative stress.^[36–38]^ This stress can damage DNA, cell membranes, and proteins. Scientific evidence has described potential mechanisms linking iron status to liver damage, which may contribute to an imbalance in M1/M2 macrophage polarization and result in fibrosis in MASLD.^[39,40]^

Second, recent in vitro and animal studies have shown that serum ferritin levels are positively associated with liver fat accumulation and negatively associated with the abundance of the families *Micrococcaceae*, *Candida*, and *Bacteroides*. This suggests that iron status may influence gut microbiota, thereby affecting the development of MASLD.^[41]^ Higher ferritin levels were also negatively correlated with serum zinc levels.^[42]^ Zinc is pivotal in the production and signaling of various inflammatory cytokines in multiple cell types,^[43]^ and zinc deficiency is considered a cause of liver fibrosis.^[44]^ In addition, inflammatory mediators can increase plasma viscosity, promote red blood cell aggregation, and enhance platelet activation, leading to heightened resistance to blood flow in organs and compromised microcirculation.^[45]^ Research involving animal models of fatty liver has demonstrated that fat accumulation decreases hepatic blood flow and microcirculation within the parenchyma, further exacerbating liver damage.^[46]^ Beyond being a biomarker, Hb represents a potentially modifiable risk factor. Targeting its underlying physiological drivers – such as reducing dietary heme-iron, improving insulin sensitivity through exercise, or considering phlebotomy in specific iron-overload cases – can mitigate Hb-associated metabolic derangements, offering novel preventative strategies for MASLD.

The primary advantages of this study lie in its use of large-scale cross-sectional data and rigorous weighting methods to ensure the generalizability of the results across the entire US population. Moreover, the inclusion of extensive genetic data and MR analysis offers significant advantages over traditional observational studies by controlling for confounding factors. Although our MR analysis helps mitigate reverse causality at the genetic level, the complex physiological interplay between liver steatosis and hematopoiesis warrants consideration. Specifically, the chronic low-grade inflammation inherent to MASLD can alter systemic iron homeostasis and suppress erythropoiesis, which may consequently influence circulating Hb levels. Therefore, a bidirectional physiological effect cannot be entirely ruled out in clinical settings.

However, this study has some limitations. First, despite multivariate adjustments, confounding factors affecting the results may still be unaccounted for. Specifically, unmeasured variables such as menopausal status, residential altitude, pulmonary diseases, and systemic inflammation could simultaneously influence Hb and MASLD, potentially affecting the precision of our identified 12.9 g/dL threshold. Furthermore, the lack of comprehensive iron metabolism data (e.g., ferritin, transferrin saturation, and hepcidin) restricts our ability to fully validate the underlying iron-driven mechanisms. In addition, some data (e.g., medical history and smoking status) relied on self-reported information, which may introduce reporting bias. Furthermore, MR analysis can only reveal linear causal relationships and cannot exclude nonlinear relationships. Another limitation is that MR research predominantly uses samples from European populations, whereas our observational studies included diverse US populations, which might affect the generalizability of the results. Finally, the genetic GWAS data used had a comparatively small sample size, and expanding the sample size is necessary to improve the accuracy of genetic assessments. Another limitation is our reliance on US-FLI to define MASLD, rather than gold-standard liver biopsy or magnetic resonance imaging-proton density fat fraction. This noninvasive surrogate cannot precisely quantify liver fat, potentially introducing misclassification bias for mild steatosis. However, a sensitivity analysis using a more stringent cutoff (US-FLI ≥ 60) confirmed that the positive association between elevated Hb and MASLD remains robust and statistically significant.

## 5. Conclusions

Our findings revealed a significant positive correlation between Hb concentration and the incidence of MASLD, clarifying the nonlinear relationship between these 2 variables. In addition, MR analysis confirmed the genetic causality of the observed associations, which is crucial for understanding their relationships. These findings underscore the need for further investigations into the possible role of Hb in the prevention and management of MASLD.

## Acknowledgments

We sincerely thank the participants and the investigators of the NHANES, IEU, and FinnGen Consortium study for their invaluable contributions, which have provided significant support and assistance to our research endeavors.

## Author contributions

**Conceptualization:** Yang Qu, Feifei Yan.

**Data curation:** Yang Qu, Guoxing Zhou, Feifei Yan.

**Formal analysis:** Yang Qu.

**Methodology:** Guoxing Zhou.

**Visualization:** Guoxing Zhou.

**Writing – original draft:** Yang Qu.

**Writing – review & editing:** Feifei Yan.





**Figure:**
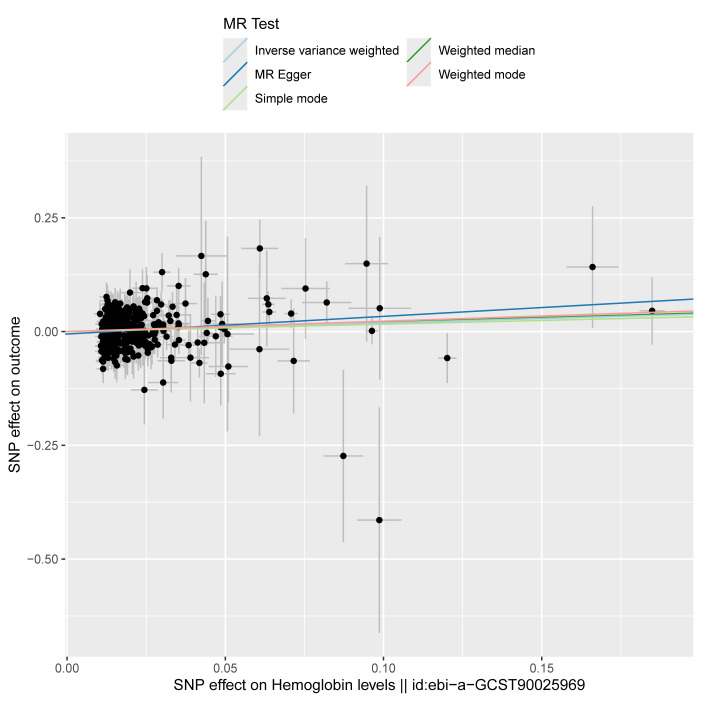


**Figure:**
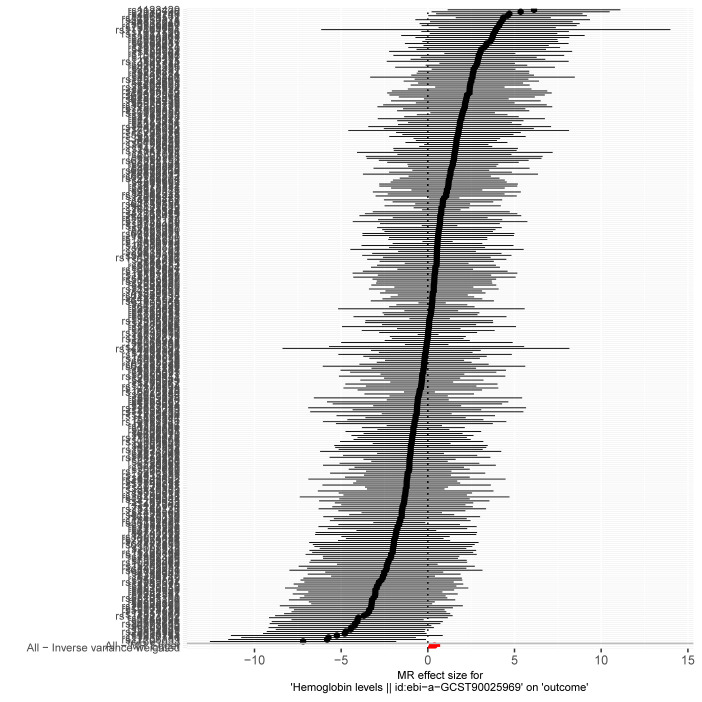


**Figure:**
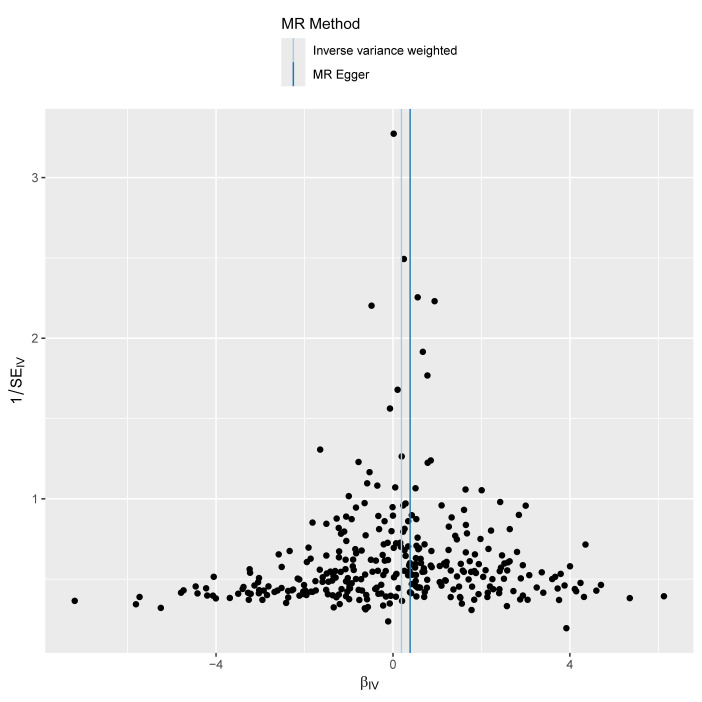


**Figure:**