# Supplementary material for: Identifying the association between hemoglobin levels and metabolic dysfunction-associated steatotic liver disease: An observational study and Mendelian randomization analysis
Source: Medicine (Baltimore). 2026 May 8;105(19):e48714. doi: 10.1097/MD.0000000000048714 (PMC13166746; doi:10.1097/MD.0000000000048714)
Supplement: Supplementary file 1 [file medi-105-e48714-s001.docx]

|  | Hb | NAFLD |
| --- | --- | --- |
| Dataset | IEU：Ebi-a-GCST90025969 | FinnGen：NAFLD |
| Sample size | 445,373 Europeans | 450,727 Europeans (2,568 cases and 448,159 controls) |

**Table S1** Detailed information about the GWAS data sources for hemoglobin and MASLD.
