# Supplementary material for: Identifying the association between hemoglobin levels and metabolic dysfunction-associated steatotic liver disease: An observational study and Mendelian randomization analysis
Source: Medicine (Baltimore). 2026 May 8;105(19):e48714. doi: 10.1097/MD.0000000000048714 (PMC13166746; doi:10.1097/MD.0000000000048714)
Supplement: Supplementary file 2 [file medi-105-e48714-s002.docx]

**Table S2** Detailed information of the 326 independent single nucleotide polymorphisms (SNPs) selected as instrumental variables.

| SNP (rsID) | Chromosome | Position | Effect Allele (EA) | Other Allele (OA) | EAF | Beta | SE | P-value | F-statistic |
| --- | --- | --- | --- | --- | --- | --- | --- | --- | --- |
| rs10023050 | 4 | 88064431 | G | A | 0.393 | 0.0297 | 0.002 | 2.50e-50 | 215.51 |
| rs10099059 | 8 | 95986972 | C | G | 0.512 | -0.0219 | 0.002 | 4.90e-29 | 122.85 |
| rs10153304 | 17 | 7721931 | A | G | 0.112 | -0.024 | 0.0033 | 5.40e-13 | 54.27 |
| rs10187311 | 2 | 47054082 | A | G | 0.06 | -0.0233 | 0.0041 | 8.00e-09 | 31.6 |
| rs10224002 | 7 | 151415041 | G | A | 0.289 | -0.0639 | 0.0022 | 3.30e-194 | 863.47 |
| rs102275 | 11 | 61557803 | C | T | 0.351 | 0.0303 | 0.0021 | 6.10e-50 | 214.79 |
| rs10245602 | 7 | 134572981 | T | C | 0.112 | -0.0186 | 0.0031 | 2.90e-10 | 35.28 |
| rs10267576 | 7 | 44870933 | C | T | 0.434 | -0.0123 | 0.002 | 3.70e-10 | 38.52 |
| rs1040360 | 21 | 16792056 | G | T | 0.301 | -0.0282 | 0.0022 | 1.70e-38 | 170.42 |
| rs10495928 | 2 | 46353166 | G | A | 0.336 | -0.0708 | 0.0021 | 1.00e-200 | 1154.81 |
| rs10509967 | 10 | 114695932 | C | A | 0.283 | 0.0138 | 0.0022 | 2.40e-11 | 39.9 |
| rs1054368 | 9 | 79791793 | T | C | 0.498 | -0.0104 | 0.002 | 3.50e-08 | 27.76 |
| rs1061338 | 19 | 33878198 | G | A | 0.261 | -0.0131 | 0.0023 | 1.90e-08 | 33.57 |
| rs10758189 | 9 | 33125804 | C | T | 0.315 | -0.0124 | 0.0021 | 2.50e-09 | 34.15 |
| rs10800598 | 1 | 198869514 | C | T | 0.638 | 0.0132 | 0.002 | 2.50e-11 | 41.85 |
| rs10850411 | 12 | 115387796 | C | T | 0.305 | -0.0122 | 0.0021 | 1.10e-08 | 32.24 |
| rs10875468 | 8 | 142245464 | C | T | 0.907 | -0.0187 | 0.0034 | 1.50e-08 | 30.12 |
| rs10878178 | 12 | 64885951 | T | C | 0.557 | 0.0121 | 0.002 | 7.50e-10 | 36.87 |
| rs10929830 | 2 | 12917172 | A | G | 0.368 | 0.0166 | 0.002 | 8.50e-16 | 66.33 |
| rs10958605 | 8 | 40053605 | A | C | 0.439 | 0.0136 | 0.002 | 2.30e-12 | 46.67 |
| rs1106766 | 12 | 57809456 | T | C | 0.24 | 0.0192 | 0.0023 | 5.60e-19 | 69.12 |
| rs1110544 | 16 | 79199110 | C | T | 0.431 | -0.0143 | 0.002 | 8.30e-12 | 51.45 |
| rs11121529 | 1 | 10271688 | G | C | 0.115 | 0.0251 | 0.0031 | 8.10e-16 | 66.05 |
| rs11160504 | 14 | 99683246 | C | T | 0.443 | 0.0121 | 0.002 | 2.30e-10 | 36.55 |
| rs11169943 | 12 | 52273248 | T | C | 0.109 | 0.0226 | 0.0032 | 3.00e-13 | 51.18 |
| rs11191424 | 10 | 104625886 | A | G | 0.329 | -0.0145 | 0.0021 | 1.50e-11 | 47.42 |
| rs1123429 | 3 | 30149009 | A | G | 0.256 | 0.0125 | 0.0023 | 2.90e-09 | 30.33 |
| rs11242732 | 6 | 2037175 | T | C | 0.444 | 0.0231 | 0.002 | 4.60e-32 | 136.12 |
| rs114922507 | 4 | 55794469 | T | C | 0.045 | -0.0304 | 0.0048 | 2.00e-10 | 40.45 |
| rs114992356 | 6 | 25983373 | T | G | 0.01 | 0.0988 | 0.0099 | 6.10e-24 | 99.55 |
| rs11538 | 22 | 18220831 | G | A | 0.172 | 0.0157 | 0.0026 | 2.10e-09 | 35.43 |
| rs11556924 | 7 | 129663496 | T | C | 0.388 | -0.0159 | 0.0021 | 3.30e-16 | 60.25 |
| rs11586825 | 1 | 217421415 | C | T | 0.12 | 0.0274 | 0.003 | 6.70e-20 | 82.7 |
| rs11677172 | 2 | 121991967 | T | C | 0.139 | 0.0289 | 0.0028 | 3.70e-24 | 103.73 |
| rs117864261 | 7 | 100363998 | T | C | 0.016 | -0.0424 | 0.008 | 5.70e-09 | 28.39 |
| rs1182933 | 12 | 121454622 | T | C | 0.297 | 0.023 | 0.0022 | 2.90e-27 | 112.72 |
| rs11857366 | 15 | 99263801 | G | A | 0.61 | 0.012 | 0.002 | 6.60e-09 | 34.68 |
| rs11879672 | 19 | 41328365 | T | C | 0.039 | -0.0716 | 0.0051 | 3.80e-46 | 195.61 |
| rs11919078 | 3 | 37996268 | A | G | 0.135 | 0.0276 | 0.0029 | 3.00e-21 | 91.43 |
| rs11933919 | 4 | 48600773 | C | G | 0.487 | -0.0134 | 0.002 | 3.80e-11 | 44.86 |
| rs12045097 | 1 | 17345877 | G | A | 0.197 | -0.0144 | 0.0025 | 5.70e-09 | 34.11 |
| rs12066445 | 1 | 151740420 | A | G | 0.32 | 0.0141 | 0.0021 | 2.30e-11 | 44.83 |
| rs1209384 | 1 | 43765089 | G | A | 0.609 | -0.0195 | 0.002 | 1.70e-22 | 93.48 |
| rs12192672 | 6 | 7229619 | A | G | 0.295 | 0.0279 | 0.0022 | 2.30e-38 | 164.9 |
| rs12206204 | 6 | 26116982 | T | C | 0.017 | 0.0753 | 0.0077 | 2.30e-22 | 96.56 |
| rs12259037 | 10 | 30300079 | A | G | 0.416 | 0.0116 | 0.002 | 1.30e-09 | 33.38 |
| rs123698 | 19 | 807442 | C | G | 0.604 | 0.0284 | 0.002 | 8.80e-47 | 197.38 |
| rs1239682 | 13 | 51165494 | T | C | 0.535 | -0.0145 | 0.002 | 2.50e-14 | 53.54 |
| rs12424336 | 12 | 24156900 | G | A | 0.274 | 0.0129 | 0.0022 | 4.90e-10 | 33.79 |
| rs12435857 | 14 | 64723525 | A | G | 0.448 | -0.0221 | 0.002 | 2.80e-30 | 123.06 |
| rs12445159 | 16 | 88962880 | T | C | 0.15 | 0.015 | 0.0028 | 2.40e-08 | 29.3 |
| rs1264081 | 1 | 230311176 | G | A | 0.158 | 0.0142 | 0.0027 | 2.30e-08 | 27.63 |
| rs12768957 | 10 | 82187293 | A | G | 0.135 | -0.0191 | 0.0029 | 7.50e-12 | 43.64 |
| rs12811512 | 12 | 13046211 | T | C | 0.152 | -0.03 | 0.0027 | 1.70e-28 | 119.13 |
| rs12881869 | 14 | 50923249 | T | C | 0.069 | -0.0287 | 0.004 | 4.20e-14 | 52.72 |
| rs12889267 | 14 | 21542766 | G | A | 0.166 | -0.0253 | 0.0027 | 4.40e-20 | 85.24 |
| rs1292061 | 17 | 57911230 | G | A | 0.555 | 0.0205 | 0.002 | 1.30e-24 | 106.26 |
| rs12945870 | 17 | 7737331 | T | C | 0.568 | -0.0198 | 0.002 | 5.60e-23 | 98.67 |
| rs12986413 | 19 | 2170954 | T | A | 0.461 | 0.0307 | 0.002 | 5.00e-56 | 239.17 |
| rs12987661 | 2 | 69813458 | C | T | 0.125 | 0.0202 | 0.003 | 6.50e-13 | 46.32 |
| rs13107325 | 4 | 103188709 | T | C | 0.075 | 0.0438 | 0.0038 | 1.40e-31 | 131.93 |
| rs13146355 | 4 | 77412140 | A | G | 0.455 | 0.0248 | 0.002 | 1.20e-37 | 155.85 |
| rs13179034 | 5 | 154059487 | A | C | 0.11 | 0.0246 | 0.0032 | 2.70e-15 | 60.6 |
| rs13223756 | 7 | 116397572 | G | A | 0.191 | 0.0138 | 0.0025 | 3.40e-08 | 29.78 |
| rs1327235 | 20 | 10969030 | G | A | 0.476 | 0.0119 | 0.002 | 5.50e-09 | 35.61 |
| rs13291790 | 9 | 13970372 | G | A | 0.216 | -0.0279 | 0.0024 | 2.30e-32 | 135.97 |
| rs13306780 | 17 | 42329004 | C | A | 0.696 | -0.0257 | 0.0022 | 6.40e-33 | 139.26 |
| rs140869992 | 16 | 2158570 | A | G | 0.016 | -0.0507 | 0.0083 | 4.40e-10 | 37.59 |
| rs1435166 | 1 | 231502310 | C | T | 0.621 | 0.0255 | 0.002 | 5.90e-36 | 154.96 |
| rs1464093 | 8 | 76474058 | G | A | 0.192 | -0.0199 | 0.0025 | 1.20e-15 | 62.97 |
| rs1472267 | 6 | 166185025 | G | A | 0.375 | 0.0129 | 0.002 | 6.70e-11 | 40.2 |
| rs1479560 | 5 | 76468398 | T | G | 0.144 | 0.0187 | 0.0028 | 1.80e-11 | 44.31 |
| rs1505991 | 5 | 40597754 | A | G | 0.883 | -0.0226 | 0.0031 | 2.30e-13 | 54.08 |
| rs151245 | 18 | 24449049 | T | G | 0.603 | -0.0117 | 0.002 | 2.00e-09 | 32.94 |
| rs1530559 | 2 | 135755629 | G | A | 0.398 | -0.0149 | 0.002 | 6.00e-14 | 52.81 |
| rs1532085 | 15 | 58683366 | G | A | 0.614 | 0.0159 | 0.002 | 1.10e-16 | 61.34 |
| rs1555576 | 13 | 29226217 | T | C | 0.343 | -0.0203 | 0.0021 | 1.40e-23 | 94.67 |
| rs1658457 | 10 | 60302716 | C | T | 0.543 | -0.0165 | 0.002 | 5.10e-18 | 69.48 |
| rs1664781 | 5 | 53276301 | A | G | 0.694 | 0.0128 | 0.0021 | 2.60e-09 | 35.51 |
| rs16844790 | 2 | 160968628 | G | A | 0.18 | -0.014 | 0.0026 | 4.80e-08 | 29.82 |
| rs16874052 | 4 | 23736523 | G | A | 0.047 | 0.0486 | 0.0047 | 2.40e-26 | 108.54 |
| rs16926246 | 10 | 71093392 | T | C | 0.13 | 0.1201 | 0.0029 | 1.00e-200 | 1674.39 |
| rs16942887 | 16 | 67928042 | A | G | 0.115 | 0.0353 | 0.0031 | 1.50e-29 | 129.82 |
| rs16956273 | 15 | 72117102 | A | G | 0.237 | -0.0151 | 0.0023 | 4.20e-12 | 42.21 |
| rs17168594 | 7 | 15048776 | C | T | 0.531 | -0.0123 | 0.002 | 1.50e-09 | 38.48 |
| rs17206359 | 4 | 157635103 | G | A | 0.321 | 0.0114 | 0.0021 | 1.90e-08 | 28.9 |
| rs172629 | 4 | 55407762 | G | C | 0.126 | -0.0485 | 0.003 | 1.90e-61 | 264.53 |
| rs17306779 | 10 | 5242164 | G | A | 0.235 | 0.0135 | 0.0023 | 1.20e-08 | 33.07 |
| rs1741315 | 20 | 4155948 | A | G | 0.435 | 0.0113 | 0.002 | 4.70e-08 | 31.77 |
| rs17437781 | 2 | 165424837 | G | A | 0.535 | 0.011 | 0.002 | 2.20e-08 | 30.88 |
| rs17850433 | 21 | 45746102 | C | T | 0.012 | -0.0608 | 0.0095 | 4.90e-11 | 41.04 |
| rs1800562 | 6 | 26093141 | A | G | 0.077 | 0.1849 | 0.004 | 1.00e-200 | 2119.82 |
| rs1800588 | 15 | 58723675 | T | C | 0.216 | -0.023 | 0.0024 | 8.50e-23 | 91.84 |
| rs1800961 | 20 | 43042364 | T | C | 0.031 | 0.0609 | 0.0058 | 5.70e-26 | 109 |
| rs181207 | 16 | 28513530 | T | C | 0.325 | -0.0118 | 0.0021 | 6.10e-09 | 30.38 |
| rs181673 | 10 | 119388528 | C | A | 0.539 | 0.0126 | 0.002 | 2.60e-10 | 40.68 |
| rs1864357 | 9 | 114812743 | A | G | 0.198 | -0.0136 | 0.0025 | 1.60e-08 | 30.35 |
| rs1893990 | 11 | 111202486 | T | C | 0.463 | 0.0157 | 0.002 | 5.30e-15 | 62.75 |
| rs194742 | 14 | 69287483 | C | T | 0.835 | -0.0146 | 0.0027 | 3.20e-08 | 29.79 |
| rs1987070 | 2 | 46237446 | A | C | 0.262 | 0.019 | 0.0022 | 1.10e-17 | 71.33 |
| rs2007538 | 5 | 90374713 | C | T | 0.105 | -0.0182 | 0.0032 | 1.30e-08 | 32.06 |
| rs2008843 | 21 | 16534968 | C | T | 0.219 | -0.0243 | 0.0024 | 5.40e-25 | 102.87 |
| rs2016942 | 11 | 8938787 | G | A | 0.442 | -0.0225 | 0.002 | 1.30e-29 | 124.38 |
| rs2035546 | 12 | 25187871 | G | A | 0.268 | 0.0122 | 0.0022 | 3.40e-08 | 29.92 |
| rs2047866 | 15 | 76136194 | T | C | 0.524 | -0.0305 | 0.002 | 4.30e-53 | 233.27 |
| rs2052285 | 16 | 51188432 | A | G | 0.609 | -0.0166 | 0.002 | 3.90e-16 | 66.92 |
| rs2059404 | 12 | 46215163 | A | G | 0.489 | 0.0148 | 0.002 | 5.40e-14 | 55.17 |
| rs2066432 | 6 | 43934662 | T | C | 0.49 | 0.0112 | 0.002 | 5.80e-09 | 32.42 |
| rs2076148 | 20 | 39788855 | G | A | 0.478 | 0.0131 | 0.002 | 7.10e-12 | 42.99 |
| rs2093380 | 22 | 37343000 | A | C | 0.693 | -0.0163 | 0.0021 | 2.60e-14 | 57.55 |
| rs2121074 | 14 | 76675452 | G | A | 0.508 | 0.0151 | 0.002 | 3.00e-14 | 58.27 |
| rs217181 | 16 | 72114002 | T | C | 0.193 | -0.021 | 0.0025 | 3.60e-17 | 70.19 |
| rs2184540 | 9 | 93801208 | A | G | 0.236 | 0.0184 | 0.0023 | 3.10e-16 | 62.48 |
| rs2257115 | 3 | 121643303 | A | T | 0.458 | -0.0166 | 0.002 | 7.80e-17 | 67.92 |
| rs2275426 | 1 | 46487552 | A | G | 0.431 | -0.0277 | 0.002 | 9.50e-46 | 192.86 |
| rs2277222 | 10 | 13494594 | C | T | 0.3 | 0.0137 | 0.0022 | 2.10e-11 | 39.9 |
| rs2278921 | 4 | 69362301 | G | T | 0.646 | -0.0115 | 0.0021 | 8.70e-09 | 29.53 |
| rs2280364 | 15 | 78585106 | T | C | 0.11 | -0.0272 | 0.0032 | 8.40e-19 | 72.87 |
| rs2284934 | 2 | 207998800 | T | C | 0.668 | -0.0169 | 0.0021 | 3.20e-16 | 64.75 |
| rs2285333 | 7 | 91667692 | G | T | 0.375 | -0.015 | 0.0021 | 2.00e-13 | 52.32 |
| rs2293888 | 8 | 116522025 | T | C | 0.218 | 0.0223 | 0.0024 | 1.30e-20 | 86.45 |
| rs2298428 | 22 | 21982892 | T | C | 0.177 | -0.0185 | 0.0026 | 1.60e-12 | 49.38 |
| rs2307088 | 3 | 14942024 | C | T | 0.564 | -0.0168 | 0.002 | 7.20e-18 | 70.5 |
| rs2358182 | 4 | 148978406 | T | C | 0.6 | -0.0157 | 0.002 | 6.20e-16 | 60.52 |
| rs2397326 | 10 | 3797577 | G | A | 0.506 | 0.0141 | 0.002 | 1.80e-12 | 50.55 |
| rs2403058 | 14 | 102964654 | G | A | 0.648 | 0.0122 | 0.0021 | 3.60e-09 | 34.04 |
| rs241812 | 6 | 100890991 | G | A | 0.308 | 0.0136 | 0.0021 | 1.30e-10 | 40.85 |
| rs245170 | 5 | 127225607 | G | A | 0.241 | -0.0164 | 0.0023 | 1.40e-13 | 50.21 |
| rs246221 | 16 | 16138322 | C | T | 0.298 | -0.0117 | 0.0022 | 1.90e-08 | 28.94 |
| rs258813 | 5 | 142674690 | A | G | 0.327 | -0.0133 | 0.0021 | 1.70e-10 | 39.46 |
| rs2612584 | 18 | 43085771 | T | C | 0.914 | 0.0242 | 0.0035 | 2.10e-12 | 47.17 |
| rs2648861 | 8 | 129059568 | G | A | 0.577 | 0.0131 | 0.002 | 8.90e-12 | 42.95 |
| rs266085 | 10 | 44874253 | T | C | 0.334 | 0.015 | 0.0021 | 5.50e-12 | 49.96 |
| rs2696815 | 16 | 86302343 | G | T | 0.157 | -0.0154 | 0.0027 | 1.40e-08 | 32.25 |
| rs2748427 | 17 | 76121864 | G | A | 0.217 | 0.0199 | 0.0024 | 1.20e-16 | 68.18 |
| rs2834297 | 21 | 35282838 | G | T | 0.465 | 0.0199 | 0.002 | 9.30e-25 | 99.95 |
| rs2835431 | 21 | 38048839 | C | T | 0.293 | -0.0246 | 0.0022 | 1.40e-31 | 127.99 |
| rs2836878 | 21 | 40465534 | A | G | 0.267 | 0.0168 | 0.0022 | 1.10e-13 | 56.02 |
| rs28439143 | 17 | 43987723 | A | G | 0.225 | 0.0445 | 0.0024 | 3.00e-84 | 352.61 |
| rs28454947 | 17 | 79769466 | C | T | 0.199 | -0.0157 | 0.0026 | 1.30e-10 | 37.56 |
| rs2847281 | 18 | 12821593 | G | A | 0.399 | -0.0157 | 0.002 | 1.60e-15 | 60.19 |
| rs2858813 | 13 | 33723857 | G | A | 0.494 | -0.0142 | 0.002 | 4.50e-13 | 51.48 |
| rs2878517 | 1 | 154961458 | G | A | 0.029 | -0.0631 | 0.0059 | 9.10e-29 | 113.38 |
| rs2930796 | 10 | 126485684 | G | A | 0.238 | 0.0128 | 0.0023 | 2.10e-08 | 30.3 |
| rs2943659 | 2 | 227125254 | A | G | 0.636 | 0.0145 | 0.002 | 8.30e-13 | 50.47 |
| rs2970871 | 4 | 23890582 | C | T | 0.558 | -0.0128 | 0.002 | 1.40e-10 | 40.91 |
| rs2971868 | 2 | 234329549 | C | T | 0.614 | 0.0108 | 0.002 | 2.00e-08 | 28.65 |
| rs2974354 | 8 | 42413943 | G | A | 0.592 | 0.0257 | 0.002 | 2.10e-38 | 163.08 |
| rs3118625 | 9 | 131873256 | C | A | 0.3 | -0.0122 | 0.0022 | 4.90e-09 | 31.48 |
| rs333947 | 1 | 110470764 | A | G | 0.15 | 0.0168 | 0.0028 | 1.20e-09 | 37.27 |
| rs34001486 | 4 | 115515948 | G | A | 0.131 | -0.02 | 0.0029 | 1.80e-12 | 46.61 |
| rs34651 | 5 | 72144005 | T | C | 0.916 | 0.0352 | 0.0036 | 6.10e-23 | 94.24 |
| rs346544 | 19 | 44256205 | C | T | 0.22 | 0.0246 | 0.0024 | 6.10e-25 | 105.64 |
| rs34877685 | 6 | 26017598 | A | G | 0.027 | 0.0987 | 0.007 | 7.10e-46 | 198.06 |
| rs34880730 | 5 | 121748004 | G | A | 0.349 | -0.0123 | 0.0021 | 3.20e-09 | 35.31 |
| rs34881325 | 9 | 2622134 | T | C | 0.374 | 0.0231 | 0.0021 | 2.90e-28 | 124.76 |
| rs34924831 | 3 | 143863020 | G | A | 0.317 | 0.0144 | 0.0021 | 1.30e-11 | 46.22 |
| rs34952318 | 20 | 11177055 | A | G | 0.05 | 0.0346 | 0.0046 | 2.40e-15 | 56.67 |
| rs35407591 | 12 | 2517887 | A | G | 0.372 | 0.0284 | 0.002 | 1.80e-46 | 193.16 |
| rs357282 | 5 | 38869035 | T | G | 0.128 | -0.0238 | 0.003 | 1.30e-15 | 64.1 |
| rs36104352 | 8 | 23377604 | C | A | 0.12 | 0.018 | 0.003 | 5.70e-10 | 35.15 |
| rs36571 | 22 | 30200713 | A | G | 0.093 | 0.0253 | 0.0034 | 1.60e-13 | 54.74 |
| rs3740689 | 11 | 47380593 | A | G | 0.584 | 0.0162 | 0.002 | 3.80e-15 | 62.82 |
| rs3747587 | 16 | 4674954 | G | C | 0.811 | 0.0154 | 0.0026 | 1.20e-10 | 36.54 |
| rs3760994 | 19 | 1435771 | A | G | 0.497 | 0.0147 | 0.002 | 2.10e-13 | 55.29 |
| rs3765522 | 1 | 183077618 | T | C | 0.548 | 0.012 | 0.002 | 3.20e-09 | 35.66 |
| rs3772219 | 3 | 56771251 | C | A | 0.325 | -0.0279 | 0.0021 | 1.00e-42 | 172.43 |
| rs3780474 | 9 | 32425676 | G | T | 0.36 | 0.0184 | 0.0021 | 2.60e-19 | 80.19 |
| rs3791020 | 1 | 173813197 | A | G | 0.238 | -0.015 | 0.0023 | 9.20e-11 | 42.33 |
| rs3809627 | 16 | 30103160 | A | C | 0.402 | 0.0228 | 0.002 | 1.30e-30 | 127.43 |
| rs3810291 | 19 | 47569003 | A | G | 0.675 | -0.0197 | 0.0021 | 1.20e-21 | 86.56 |
| rs3811444 | 1 | 248039451 | T | C | 0.333 | 0.0229 | 0.0021 | 4.00e-27 | 117.99 |
| rs3816048 | 19 | 4475606 | C | T | 0.562 | 0.0286 | 0.002 | 7.20e-48 | 199.76 |
| rs3891585 | 2 | 66756976 | G | A | 0.606 | 0.013 | 0.002 | 3.80e-10 | 41.4 |
| rs3934103 | 3 | 41977214 | T | C | 0.837 | -0.0192 | 0.0027 | 1.20e-13 | 50.7 |
| rs4075958 | 5 | 176784512 | A | G | 0.263 | 0.0256 | 0.0022 | 2.50e-31 | 130.46 |
| rs4103 | 13 | 111098226 | T | C | 0.54 | 0.0109 | 0.002 | 1.10e-08 | 29.7 |
| rs412181 | 20 | 57298627 | A | G | 0.526 | -0.0111 | 0.002 | 4.90e-08 | 31.23 |
| rs41267675 | 6 | 56600121 | A | G | 0.168 | 0.0168 | 0.0027 | 4.30e-11 | 39.55 |
| rs41278174 | 16 | 16259596 | A | G | 0.027 | 0.051 | 0.0063 | 3.50e-16 | 66.38 |
| rs4245633 | 1 | 31593640 | T | C | 0.13 | 0.0174 | 0.0029 | 4.90e-09 | 35.22 |
| rs4247303 | 2 | 85554080 | A | G | 0.478 | -0.0113 | 0.002 | 6.50e-09 | 32.38 |
| rs4282786 | 1 | 214176869 | A | G | 0.262 | 0.0384 | 0.0022 | 1.90e-67 | 295.37 |
| rs4366668 | 15 | 65903398 | A | G | 0.428 | -0.0171 | 0.002 | 2.60e-17 | 72.96 |
| rs4394621 | 1 | 66165836 | A | G | 0.817 | -0.0165 | 0.0025 | 6.30e-11 | 41.93 |
| rs445036 | 8 | 81408409 | C | T | 0.29 | -0.0167 | 0.0022 | 4.50e-13 | 58.63 |
| rs4464148 | 18 | 46459032 | C | T | 0.302 | -0.0198 | 0.0022 | 5.80e-20 | 83.49 |
| rs448378 | 3 | 169100899 | A | G | 0.527 | 0.0158 | 0.002 | 4.50e-16 | 64.26 |
| rs45565742 | 14 | 65510971 | T | C | 0.138 | 0.0269 | 0.0029 | 1.40e-20 | 86.34 |
| rs4573764 | 12 | 4330215 | T | C | 0.209 | -0.019 | 0.0024 | 2.40e-16 | 61.35 |
| rs4574537 | 5 | 137419728 | C | T | 0.316 | 0.0127 | 0.0021 | 1.30e-08 | 35.1 |
| rs4594362 | 19 | 50059982 | G | C | 0.097 | 0.0212 | 0.0034 | 6.10e-10 | 39.5 |
| rs460879 | 16 | 89712889 | T | C | 0.427 | 0.0239 | 0.002 | 1.90e-33 | 140.03 |
| rs4638745 | 2 | 29093803 | G | A | 0.428 | 0.0118 | 0.002 | 1.20e-08 | 35.19 |
| rs4646018 | 1 | 15840374 | C | T | 0.527 | 0.0126 | 0.002 | 6.10e-11 | 41.3 |
| rs464605 | 5 | 55807370 | T | C | 0.746 | 0.0172 | 0.0023 | 1.30e-15 | 57.79 |
| rs4656020 | 1 | 88646891 | A | G | 0.673 | -0.0134 | 0.0021 | 6.80e-11 | 40.75 |
| rs4743035 | 9 | 109637284 | G | A | 0.199 | 0.0147 | 0.0025 | 2.00e-09 | 35.4 |
| rs4760682 | 12 | 48512285 | A | C | 0.807 | -0.0485 | 0.0025 | 2.50e-84 | 366.79 |
| rs477992 | 1 | 120257576 | G | A | 0.679 | 0.0146 | 0.0021 | 2.00e-12 | 48.1 |
| rs4792555 | 17 | 14817763 | G | A | 0.744 | 0.0165 | 0.0023 | 2.40e-12 | 52.69 |
| rs4795457 | 17 | 27087929 | T | C | 0.203 | 0.0207 | 0.0025 | 2.40e-17 | 71.05 |
| rs4805801 | 19 | 33183812 | T | C | 0.188 | -0.0236 | 0.0025 | 2.60e-21 | 86.61 |
| rs4811074 | 20 | 49100715 | T | C | 0.321 | 0.02 | 0.0021 | 6.90e-23 | 88.85 |
| rs4812048 | 20 | 57587771 | T | C | 0.182 | -0.0152 | 0.0026 | 9.90e-10 | 34.88 |
| rs4841132 | 8 | 9183596 | G | A | 0.909 | -0.0352 | 0.0037 | 4.90e-23 | 93.06 |
| rs4883569 | 12 | 133102462 | A | G | 0.273 | 0.015 | 0.0022 | 5.10e-12 | 45.3 |
| rs4909945 | 11 | 10673739 | C | T | 0.688 | 0.0165 | 0.0021 | 8.40e-15 | 59.1 |
| rs4923457 | 11 | 27648580 | T | A | 0.201 | 0.0159 | 0.0025 | 4.20e-11 | 41.57 |
| rs4951070 | 1 | 203652698 | C | T | 0.902 | -0.0413 | 0.0033 | 8.60e-35 | 155.44 |
| rs499974 | 11 | 75455021 | A | C | 0.157 | 0.0225 | 0.0027 | 2.80e-17 | 68.65 |
| rs5024246 | 1 | 3318714 | A | G | 0.353 | -0.0117 | 0.0021 | 1.50e-08 | 32.58 |
| rs510803 | 1 | 28559574 | G | C | 0.291 | 0.0122 | 0.0022 | 3.50e-09 | 31.58 |
| rs5369 | 6 | 12294258 | G | A | 0.874 | -0.0243 | 0.003 | 3.70e-16 | 64.87 |
| rs55707100 | 15 | 43820717 | T | C | 0.026 | -0.0873 | 0.0063 | 2.10e-44 | 192.2 |
| rs55728759 | 1 | 12660420 | C | T | 0.357 | 0.0132 | 0.0021 | 2.30e-10 | 40.81 |
| rs56146525 | 5 | 442571 | C | A | 0.404 | 0.0177 | 0.002 | 2.80e-20 | 76.25 |
| rs56199187 | 1 | 161520931 | T | C | 0.119 | 0.0326 | 0.003 | 2.30e-28 | 115.61 |
| rs56250311 | 1 | 232888003 | G | A | 0.092 | -0.0199 | 0.0034 | 9.30e-10 | 34.15 |
| rs56352102 | 11 | 10268593 | T | C | 0.184 | -0.0418 | 0.0025 | 1.90e-64 | 269.61 |
| rs572802 | 1 | 172170918 | T | C | 0.42 | -0.0188 | 0.002 | 6.10e-22 | 88.65 |
| rs573455 | 11 | 117267884 | G | A | 0.531 | 0.014 | 0.002 | 1.90e-11 | 48.53 |
| rs5750383 | 22 | 37520225 | G | A | 0.523 | -0.0207 | 0.002 | 1.10e-25 | 108.86 |
| rs5758896 | 22 | 43115576 | C | T | 0.595 | 0.0261 | 0.002 | 6.00e-39 | 167.45 |
| rs58433687 | 3 | 58223292 | C | T | 0.66 | 0.0167 | 0.0021 | 2.70e-17 | 64.55 |
| rs586194 | 2 | 219610433 | G | A | 0.479 | -0.0213 | 0.002 | 3.70e-26 | 114.55 |
| rs592423 | 6 | 139840693 | C | A | 0.554 | -0.0152 | 0.002 | 1.10e-14 | 59.41 |
| rs60290266 | 3 | 12348835 | A | G | 0.241 | -0.0272 | 0.0023 | 1.50e-32 | 139.08 |
| rs6051217 | 20 | 26178568 | G | T | 0.215 | -0.0159 | 0.0024 | 2.00e-11 | 43.37 |
| rs6055971 | 20 | 8627642 | T | C | 0.229 | -0.0132 | 0.0024 | 7.90e-09 | 31.58 |
| rs6070139 | 20 | 56119037 | G | A | 0.599 | 0.0196 | 0.002 | 1.40e-24 | 94.11 |
| rs61033544 | 2 | 112278539 | A | G | 0.123 | -0.0202 | 0.003 | 2.20e-12 | 45.54 |
| rs61177850 | 2 | 177736333 | T | C | 0.549 | -0.0119 | 0.002 | 1.50e-09 | 36.3 |
| rs61739556 | 7 | 100205706 | C | G | 0.016 | -0.1661 | 0.0082 | 2.60e-92 | 408.31 |
| rs62022864 | 15 | 86165693 | C | G | 0.137 | 0.0182 | 0.0029 | 2.60e-10 | 39.93 |
| rs62032881 | 16 | 354287 | T | C | 0.161 | -0.0152 | 0.0027 | 2.20e-08 | 31.65 |
| rs62092152 | 18 | 42808945 | G | A | 0.123 | 0.0208 | 0.003 | 1.40e-11 | 47.46 |
| rs62398471 | 5 | 176980904 | A | G | 0.29 | -0.014 | 0.0022 | 1.60e-10 | 39.47 |
| rs62401198 | 6 | 43801654 | T | C | 0.157 | -0.0352 | 0.0027 | 5.80e-40 | 168.99 |
| rs62431456 | 7 | 652741 | A | G | 0.598 | 0.0227 | 0.002 | 1.80e-30 | 127.7 |
| rs62452196 | 7 | 25473826 | G | A | 0.083 | -0.0219 | 0.0036 | 3.20e-10 | 37.68 |
| rs62482245 | 7 | 100251369 | A | G | 0.036 | -0.047 | 0.0053 | 5.20e-20 | 78.84 |
| rs6421984 | 11 | 305619 | C | T | 0.513 | 0.0108 | 0.002 | 2.80e-08 | 29.87 |
| rs6426749 | 1 | 22711473 | C | G | 0.177 | 0.021 | 0.0026 | 1.40e-17 | 66.73 |
| rs6430059 | 2 | 145228701 | G | A | 0.689 | 0.0125 | 0.0021 | 5.10e-09 | 34.46 |
| rs6478799 | 9 | 130630430 | G | A | 0.076 | 0.0374 | 0.0037 | 9.60e-26 | 100.11 |
| rs6504340 | 17 | 46617019 | G | A | 0.786 | 0.0191 | 0.0024 | 7.50e-16 | 62.9 |
| rs653178 | 12 | 112007756 | T | C | 0.516 | -0.0636 | 0.002 | 1.00e-200 | 1037.02 |
| rs6546859 | 2 | 73842055 | A | C | 0.236 | 0.0146 | 0.0023 | 6.50e-10 | 39.56 |
| rs6587235 | 20 | 61024823 | G | A | 0.733 | -0.0178 | 0.0022 | 7.60e-17 | 63.33 |
| rs6602910 | 13 | 114549015 | C | T | 0.371 | 0.0197 | 0.0021 | 3.20e-22 | 92.11 |
| rs6664244 | 1 | 40374386 | C | T | 0.391 | -0.0117 | 0.002 | 3.20e-09 | 33.37 |
| rs6667255 | 1 | 3711689 | C | T | 0.241 | 0.0269 | 0.0023 | 1.20e-31 | 136.39 |
| rs66782572 | 3 | 52567617 | G | A | 0.534 | -0.0234 | 0.002 | 2.70e-33 | 138.39 |
| rs6682947 | 1 | 213069783 | G | A | 0.682 | 0.0157 | 0.0021 | 3.30e-13 | 55.11 |
| rs6683445 | 1 | 16368403 | A | C | 0.533 | 0.0223 | 0.002 | 2.00e-29 | 127.04 |
| rs6703350 | 1 | 33732772 | T | G | 0.504 | 0.0125 | 0.002 | 1.30e-10 | 40.29 |
| rs6738560 | 2 | 145634153 | A | G | 0.702 | -0.0255 | 0.0022 | 4.20e-31 | 140.32 |
| rs6805563 | 3 | 69879323 | A | G | 0.431 | 0.0212 | 0.002 | 6.20e-29 | 113.73 |
| rs6821756 | 4 | 187751550 | A | G | 0.926 | -0.0233 | 0.0038 | 1.40e-09 | 38.12 |
| rs6848130 | 4 | 81157098 | C | T | 0.281 | 0.013 | 0.0022 | 9.40e-10 | 35.03 |
| rs6900852 | 6 | 126223073 | A | G | 0.561 | 0.0147 | 0.002 | 1.20e-13 | 54.92 |
| rs6926552 | 6 | 16290862 | C | T | 0.206 | -0.0178 | 0.0024 | 3.50e-14 | 52.93 |
| rs6935246 | 6 | 1758012 | G | T | 0.433 | -0.012 | 0.002 | 1.30e-09 | 36.61 |
| rs6950388 | 7 | 1270699 | A | G | 0.796 | -0.0321 | 0.0025 | 5.30e-41 | 171.32 |
| rs699946 | 6 | 43732669 | G | A | 0.184 | -0.0152 | 0.0025 | 6.50e-10 | 35.84 |
| rs7026695 | 9 | 4107030 | C | T | 0.242 | -0.0152 | 0.0023 | 1.40e-10 | 43.5 |
| rs7081609 | 10 | 88939962 | C | T | 0.841 | 0.0207 | 0.0028 | 4.90e-14 | 55.26 |
| rs711818 | 2 | 176978833 | G | A | 0.265 | 0.0179 | 0.0022 | 1.40e-16 | 64.58 |
| rs71462837 | 15 | 33309755 | T | C | 0.179 | 0.0218 | 0.0026 | 2.10e-19 | 70.89 |
| rs7155504 | 14 | 36158828 | C | T | 0.088 | -0.0329 | 0.0035 | 1.00e-21 | 87.43 |
| rs7182871 | 15 | 43062936 | C | T | 0.944 | -0.0222 | 0.0043 | 3.10e-08 | 26.44 |
| rs7209653 | 17 | 19882084 | C | T | 0.295 | 0.0128 | 0.0022 | 1.40e-09 | 34.96 |
| rs7223467 | 17 | 81051887 | G | T | 0.652 | 0.0146 | 0.0021 | 3.10e-12 | 49.28 |
| rs72796138 | 2 | 23926233 | T | C | 0.13 | 0.0265 | 0.0029 | 3.70e-20 | 81.86 |
| rs73169651 | 7 | 150908886 | A | C | 0.109 | -0.0238 | 0.0032 | 3.70e-14 | 54.71 |
| rs737376 | 10 | 13735587 | A | G | 0.43 | 0.0155 | 0.002 | 1.30e-15 | 58.89 |
| rs73780221 | 6 | 142725182 | C | G | 0.028 | -0.039 | 0.006 | 3.10e-11 | 42.72 |
| rs7428617 | 3 | 194679087 | G | A | 0.508 | -0.0198 | 0.002 | 3.90e-24 | 100.24 |
| rs7464181 | 8 | 61178574 | C | T | 0.414 | 0.0137 | 0.002 | 6.30e-11 | 46.2 |
| rs7497064 | 15 | 66990159 | C | T | 0.243 | 0.0187 | 0.0023 | 2.90e-16 | 65.48 |
| rs7503383 | 17 | 58837701 | G | A | 0.918 | 0.0206 | 0.0036 | 2.50e-09 | 32.58 |
| rs752262 | 6 | 109770999 | C | G | 0.329 | 0.0126 | 0.0021 | 3.10e-09 | 35.45 |
| rs752590 | 2 | 113972945 | G | A | 0.21 | 0.0305 | 0.0024 | 1.60e-38 | 159.14 |
| rs756110 | 2 | 43426617 | T | C | 0.5 | -0.0184 | 0.002 | 1.90e-20 | 87.23 |
| rs7568408 | 2 | 242648549 | A | G | 0.446 | 0.0143 | 0.002 | 4.10e-13 | 50.47 |
| rs757721 | 7 | 100817667 | T | G | 0.909 | -0.0192 | 0.0034 | 5.10e-09 | 31.26 |
| rs75792643 | 22 | 46372968 | T | C | 0.309 | 0.0333 | 0.0022 | 9.00e-57 | 236.51 |
| rs75869289 | 2 | 203806724 | T | C | 0.127 | 0.0184 | 0.003 | 3.40e-10 | 38.7 |
| rs760077 | 1 | 155178782 | T | A | 0.606 | 0.0314 | 0.002 | 9.90e-57 | 238.35 |
| rs7601633 | 2 | 62394127 | G | A | 0.344 | -0.0114 | 0.0021 | 2.10e-08 | 30.24 |
| rs7627706 | 3 | 195753353 | C | A | 0.675 | 0.0133 | 0.0021 | 9.90e-12 | 40.02 |
| rs76643124 | 9 | 136356448 | A | G | 0.016 | 0.082 | 0.0078 | 5.60e-26 | 110.9 |
| rs77068084 | 1 | 56957612 | C | T | 0.06 | -0.0218 | 0.0041 | 2.00e-08 | 27.85 |
| rs77080560 | 5 | 111245325 | G | A | 0.076 | -0.0231 | 0.0037 | 2.00e-10 | 38.15 |
| rs7748291 | 6 | 37039564 | T | C | 0.501 | -0.0182 | 0.002 | 1.20e-20 | 85.57 |
| rs77542162 | 17 | 67081278 | G | A | 0.023 | 0.0946 | 0.0068 | 1.00e-45 | 195.22 |
| rs77685055 | 13 | 76055602 | A | G | 0.099 | 0.0182 | 0.0033 | 2.70e-08 | 29.66 |
| rs7816734 | 8 | 41547748 | A | G | 0.058 | 0.0244 | 0.0042 | 1.30e-08 | 33.06 |
| rs782722 | 3 | 66338718 | T | A | 0.364 | 0.012 | 0.0021 | 1.10e-08 | 34.21 |
| rs7855434 | 9 | 116288537 | C | T | 0.188 | -0.0216 | 0.0025 | 2.20e-17 | 73.24 |
| rs7875332 | 9 | 114927535 | C | T | 0.442 | -0.0143 | 0.002 | 2.80e-13 | 51.84 |
| rs78938703 | 6 | 27470055 | C | T | 0.04 | -0.049 | 0.0052 | 4.70e-21 | 87.14 |
| rs7954238 | 12 | 91154207 | C | T | 0.272 | 0.015 | 0.0022 | 6.00e-13 | 45.55 |
| rs797343 | 14 | 34646269 | T | C | 0.682 | 0.0165 | 0.0021 | 1.90e-15 | 59.96 |
| rs79953286 | 3 | 132226100 | G | A | 0.057 | -0.0329 | 0.0043 | 1.90e-14 | 58.34 |
| rs8009224 | 14 | 74237247 | A | G | 0.024 | 0.0433 | 0.0065 | 3.20e-11 | 44.93 |
| rs8010057 | 14 | 23506110 | T | C | 0.419 | -0.0107 | 0.002 | 3.30e-08 | 28.28 |
| rs805316 | 2 | 54133744 | C | T | 0.275 | 0.0151 | 0.0022 | 5.80e-13 | 46.67 |
| rs8074498 | 17 | 79954544 | A | T | 0.573 | 0.0112 | 0.002 | 1.60e-08 | 30.01 |
| rs837763 | 16 | 88853729 | T | C | 0.556 | -0.0441 | 0.002 | 5.70e-111 | 491.15 |
| rs855791 | 22 | 37462936 | G | A | 0.558 | 0.0963 | 0.002 | 1.00e-200 | 2266.99 |
| rs871841 | 17 | 8216468 | C | T | 0.492 | 0.0191 | 0.002 | 8.60e-23 | 87.89 |
| rs881858 | 6 | 43806609 | A | G | 0.693 | 0.0354 | 0.0021 | 2.40e-64 | 275.85 |
| rs903518 | 3 | 23336968 | G | A | 0.323 | 0.0157 | 0.0021 | 4.90e-15 | 56.07 |
| rs911028 | 6 | 166070424 | T | C | 0.719 | -0.0156 | 0.0022 | 7.20e-14 | 50.57 |
| rs9295536 | 6 | 22131929 | A | C | 0.431 | 0.019 | 0.002 | 3.80e-21 | 91.55 |
| rs9402686 | 6 | 135427817 | A | G | 0.271 | -0.0497 | 0.0022 | 9.29e-116 | 506.91 |
| rs9415106 | 10 | 77295957 | A | G | 0.715 | 0.0157 | 0.0022 | 1.00e-12 | 51.52 |
| rs9458808 | 6 | 163755252 | G | A | 0.195 | -0.0186 | 0.0025 | 2.40e-13 | 55.97 |
| rs9491696 | 6 | 127452639 | G | C | 0.493 | 0.0278 | 0.002 | 2.70e-46 | 200.54 |
| rs963837 | 11 | 30749090 | C | T | 0.453 | 0.0249 | 0.002 | 1.10e-35 | 157.18 |
| rs9649959 | 8 | 128972721 | G | A | 0.344 | -0.0181 | 0.0021 | 8.70e-19 | 75.57 |
| rs9863706 | 3 | 72437413 | T | C | 0.219 | 0.0146 | 0.0024 | 2.90e-10 | 37.39 |
| rs9895661 | 17 | 59456589 | T | C | 0.829 | -0.0341 | 0.0026 | 1.40e-39 | 167.59 |
| rs9920011 | 15 | 95690709 | G | A | 0.061 | 0.0229 | 0.0041 | 2.30e-09 | 30.76 |
